# Determinants of blood and saliva lead concentrations in adult gardeners on urban agricultural sites

**DOI:** 10.1007/s10653-021-01095-7

**Published:** 2021-10-07

**Authors:** Lindsay Bramwell, Jackie Morton, Anne-Helen Harding, Nan Lin, Jane Entwistle

**Affiliations:** 1 Population Health Sciences Institute, Newcastle University, Newcastle upon Tyne, NE2 4AX Tyne and Wear UK; 2 Present Address: Department of Geography and Environmental Sciences, Northumbria University, Ellison Building, Newcastle Upon Tyne, NE1 8ST Tyne and Wear UK; 3 Health and Safety Executive Science and Research Centre, Buxton, SK17 9JN Derbyshire UK; 4 Department of Mathematics, Physics and Electrical Engineering, Northumbria University, Ellison Building, Newcastle Upon Tyne, NE1 8ST Tyne and Wear UK

**Keywords:** Human biomonitoring, Blood lead, Saliva lead, Urban agriculture, Exposure assessment, Allotments

## Abstract

Soil Pb concentrations at urban agriculture sites (UAS) commonly exceed recommended safe levels. There is a lack of evidence regarding uptake of Pb by gardeners using such sites for food crops. Our study aimed to elucidate whether gardening in soil with raised Pb levels results in Pb body burdens of concern to health, and to assess confounding factors influencing Pb body burden. Our cross-sectional case study measured Pb in saliva and blood of UAS gardeners (n = 43), soil and produce samples from their UAS, and home tap water. Blood and saliva Pb concentrations were compared with those from non-UAS gardener controls (n = 29). A health risk threshold of 5 µg dL^−1^ blood Pb level (BLL) was selected in keeping with international guidance. Detailed surveys investigated individuals’ anthropometrics and potential Pb exposures from diet, and historic and everyday activities. Saliva was not found to be a suitable biomarker of adult Pb exposure in this context. Predictors of higher BLLs were being older, being male and eating more root vegetables and shrub fruit. Eating more green vegetables predicted a lower BLL, suggesting a protective effect against Pb uptake. UAS gardeners’ BLLs (geometric mean 1.53; range 0.6–4.1 µg dL^−1^) were not significantly higher (*p* = 0.39) than the control group (geometric mean 1.43; range 0.7–2.9 µg dL^−1^). All BLLs were below 5 µg dL^−1^ except one resulting from occupational exposure. Having paired the UAS gardeners with closely matched controls, we found Pb in UAS soils (with range 62–1300 mg kg^−1^from common urban sources) unlikely to pose an additional risk to adult health compared to their neighbours who did not access UAS. As such, other Pb sources may be the dominant factor controlling BLL.

**Supplementary Information:**

The online version contains supplementary material available at 10.1007/s10653-021-01095-7.

## Introduction

Urban soils often contain Pb concentrations exceeding recommended safe levels and significantly above the local regional background concentrations (BGS, 2012; Datko-Williams et al., 2014). Observations of raised urban soil Pb are reported globally in inner city areas with older housing and heavily trafficked roads (Filippelli & Laidlaw, 2010; Mielke et al., 2016; Resongles et al., 2021; Taylor et al., 2021; Wang et al., 2006). Soil Pb analyses on urban agriculture sites (UAS), known as allotments in the UK, frequently report samples exceeding health-based guidance values for Pb (Clark et al., 2008; Latimer et al., 2016; Mitchell et al., 2014; Pless Mulloli et al., 2004; Rouillon et al., 2017). Contamination can arise from many different sources including previous site use, such as industry or landfill, legacy Pb from gasoline emissions from vehicles or other fossil fuel burning, Pb-based paint particles, bonfires, runoff from metal surfaces, application of ash as a soil improver, pesticides, from hobbies and related artefacts (i.e. Pb solder) and natural geology (Alloway, 2004; Meharg, 2016). Regardless of the original source, soils act as a sink for diffuse urban Pb pollution, and exposure to soil and soil-derived dust is a potential exposure pathway for humans in the urban environment (Filippelli & Laidlaw, 2010). Contaminated UAS are increasingly of concern for local regulatory authorities with tensions between protecting public health from exposure to contaminated land and food, and supporting UAS gardening as a health promoting activity (Alaimo et al., 2008; Leake et al., 2009; Litt et al., 2011; Van Den Berg & Custers, 2011) that also provides low carbon footprint food, community development, and learning opportunities (Armstrong, 2000; Wakefield et al., 2007).

We have long been aware of the health consequences of Pb exposure (ATSDR, 2007; PHE, 2014). In recent years, there has been an improved understanding about the toxicological effects of Pb, especially those at lower levels of environmental exposure (Lanphear et al., 2005; NTP, 2012) and health exposure limits have been revised downwards. In the early 2000s, the generally accepted threshold level for Pb in blood was 10 µg dL^−1^ (CDC, 2019; DEFRA/EA, 2002; DEFRA, 2014a; WHO/JEFCA, 2011). In 2012, the US Centers for Disease Prevention and Control (CDC) lowered the public health action level for Pb in children’s blood to 5 µg dL^−1^ (CDC, 2019). The same adopted threshold is also used in Australia (NHMRC, 2016) and Canada (Buka & Hervouet-Zeiber, 2019). Whilst young children are most at risk from the neurological and developmental effects of Pb uptake, pregnant and lactating mothers are also of particular concern as Pb stored in bone can be mobilised during pregnancy (Gomaa et al., 2002; NTP, 2012; Téllez-Rojo et al., 2004). An increasing body of evidence suggests that even low levels of environmental Pb exposure contribute to reproductive effects (NTP, 2012), hypertension (Navas-Acien et al., 2007; Vupputuri et al., 2003), cardiovascular disease (Lanphear et al., 2018), chronic kidney disease (Ekong et al., 2006; Navas-Acien et al., 2007) and even spontaneous abortion in females (Gidlow, 2015).

Newcastle upon Tyne (population 280,200) is the regional capital of NE England (population 2,600,000) (ONS, 2016) with a history of coal mining and heavy industries, including the Pb industry. Newcastle City Council previously reported the findings of investigations and risk assessments for 28 UAS from across the city that had been identified as having raised contaminant concentrations (Bramwell & Pless-Mulloli, 2008). In an earlier study with > 400 UAS soil samples, Pb was repeatedly found to be elevated: median of 545 mg kg^−1^; 95^th^ percentile of 684 mg kg^−1^ (Pless-Mulloli et al., 2001). Several sources of Pb were proposed to explain the elevated soil Pb concentrations: (1) Pb in coal fire ash, historically brought onto sites from homes used as a soil amendment to break up heavy clay soils to aid plant growth; (2) old wooden window frames and doors with peeling Pb paint brought onto site to make cold frames and greenhouses; (3) bonfire ash from the old wooden window frames used as a soil amendment; (4) deposition from vehicle exhausts; and (5) deposition from domestic fires. Based on available toxicological guidance at the time, local authority regulators decided that, on balance, gardening activities and consumption of vegetables from these sites was a greater benefit than risk to health. However, in common with the reductions seen in many countries worldwide for the ‘low level of toxicological concern’ (LLTC) for blood Pb, a modelled LLTC of 3.5 µg dL^−1^ was adopted for the development of UK soil screening guidance (C4SL), meaning that many of these UAS sites now had Pb soil concentrations ten times higher than the new soil screening guideline value of 80 mg kg^−1^ (DEFRA, 2014b). Detailed quantitative risk assessments were undertaken for the UAS, including crop Pb analysis, and produce consumption rates (Entwistle et al., 2019); however, considerable uncertainty remains in the exposure modelling for UAS.

Blood lead levels (BLLs) are widely used as a reliable biomarker of inorganic Pb exposure (Barbosa et al., 2005), representing both recent absorption of Pb and removal of Pb stored in the bone of the body (Falq et al., 2011). Pb half-life in blood is reported to be 35 days (Rabinowitz et al., 1976; Rust et al., 1999). Zinc protoporphyrin (ZPP), commonly measured in tandem to BLL, represents Pb exposure over a longer period, typically 8–12 weeks depending on whether the exposure is at a steady-state exposure (Martin et al., 2004). Direct analysis of UAS gardeners and non-UAS gardeners BLL and ZPP can thus provide quantitative evidence of Pb exposure to corroborate and/or inform risk assessments and give confidence to the regulators who must decide if sites are suitable for use as UAS. For several pragmatic reasons and ethical considerations, our study focussed on adult UAS gardeners and their non-gardening adult neighbours from the same community (as controls). Blood sampling is typically invasive, with venous sampling (requiring trained professionals) typically the preferred method over finger-prick sampling where greater environmental contamination is potentially encountered (CDC, 1997). As such, alternative biomonitoring matrices are desirable.

Several studies have explored saliva as an alternative matrix for the biological monitoring of Pb (Barbosa et al., 2006; Costa de Almeida et al., 2009; Koh et al., 2003.; Nriagu et al., 2006; Staff et al., 2014). The use of saliva would have several potential advantages. Its collection is non-invasive, and therefore there are no concerns over discomfort to participants; collection is straightforward and cheap to carry out; sample storage and transport arrangements are less complex than those for blood; and in addition, the ethical approval for sampling is more easily obtained (Nriagu et al., 2006). It is thought that the Pb content of saliva may be related to the unbound fraction in the plasma (Nriagu et al., 2006), and as the plasma composition closely reflects that of the extracellular fluid, measuring salivary Pb may therefore indicate the level of exposure to which most bodily cells are subjected (Costa de Almeida et al., 2009).

Specific objectives of this study, the Newcastle Allotment Biomonitoring Study (NABS), were to (1) measure and compare Pb and ZPP in blood of UAS gardeners and their non-UAS gardening neighbours (the controls), (2) measure Pb levels in saliva to investigate its potential as an alternative biomonitoring matrix to blood, and (3) identify predictors of participant BLLs by comparing blood and saliva Pb concentrations with matched allotment soil concentrations, participants diets and participant characteristics and behaviours, whilst adjusting for confounders.

## Materials and methods

A study steering group made up of local and national experts in Pb exposure, exposure modelling, UAS gardening and biomonitoring was convened to inform study design and analysis. We recruited healthy adult volunteers from three UAS in Newcastle upon Tyne. The UAS were selected for their typically raised soil Pb concentrations and practical aspects around blood and saliva sample collection. Participant recruitment took place from July to September 2015 by visiting sites during their open days and allotment shows. Where possible, participant UAS gardeners recruited a neighbour or friend of the same sex and similar age, though not one who visited their UAS or with whom they shared their UAS produce, to act as their control. The control group for this study were selected to represent all Pb exposure in common with the UAS gardeners except those specifically relating to the UAS. They neither visited the UAS nor ate produce grown on them; however, 69% of the control group either did some ad hoc gardening themselves or lived with someone who did some. This took place either in their home garden, back yard or at the home of a family member, but not at a designated UAS. A control group selected from a more remote group may not reflect other shared everyday exposures. Persons requiring a regular blood Pb test for their occupation were excluded from the study. All samples were collected during September and October 2015 towards the end of the growing season when UAS gardeners BLL could be anticipated to be at their highest. Information on potentially confounding participant characteristics and behaviours was collected by questionnaire, along with additional matched environmental samples.

### Human biological samples

Blood samples (5 ml) were collected in K_2_EDTA vacutainers (purple top), by nurses at the UAS sites using venal puncture kit. Two saliva samples were also collected, using the Verisal collection device with the travel cover (Oasis Diagnostics, Vancouver, USA). One was collected before the blood sample, and the other after the blood sample. Participants held an absorbent sample collection paddle in their mouths until the saliva level indicator was reached. The samples were sent to the Health and Safety Executive’s (HSE’s) Laboratory, Buxton, UK, for analysis. Samples were stored in the refrigerator at 5 °C until analysis was undertaken. Blood samples were mixed at room temperature before analysis using standard operating procedures at HSE’s laboratory (a UKAS-accredited laboratory for this method). In brief, the samples were diluted 1 in 50 in an alkaline diluent (1% v/v ammonia, 0.1% m/v EDTA, 0.1% v/v Triton X100) and platinum was used as an internal standard. Sample analysis was performed by ICP-MS (Thermo Series 2 ICP-MS, Hemel Hempstead, UK). Certified reference materials (Levels 1 and 3 Trace Element in Blood (lot numbers 36773 and 36771) Bio-Rad Lyphocheck, Bio-Rad Laboratories) were analysed within the analysis. All blood samples analysed showed a replicate relative standard deviation of less than 2%. In addition to blood Pb concentrations, other known biomarkers for Pb exposure were determined (as routinely undertaken), i.e. ZPP and haemoglobin. These results are not reported here but available on request from the corresponding author. The analysis was undertaken in a UKAS-accredited laboratory. All certified reference and quality control materials used for all assays were within the certified reference material ranges.

To recover the saliva from the Oasis devices, the saliva paddle was inserted into an end device cap to facilitate the liquid extraction and plunged. The liquid squeezed from the paddle was then collected in an Eppendorf tube and frozen until analysed. However, for 71 of the 134 samples no liquid was collected when the paddle was pushed in the end device. For these samples, the swab paddle was extracted from the Oasis device with nylon tweezers, placed into a sample tube and centrifuged for 10 min at 4000 rpm. The spun liquid was retained in the bottom half of the device and capped and the rest discarded. Four samples had no liquid present using either method. Before analysis, the saliva samples were acid-digested (1:1 saliva/concentrated nitric acid at 100 °C for 1 h) and the cooled samples were then diluted with water and acid diluent (final dilution 1 in 25) and analysed by ICP-MS (Thermo Series 2 ICP-MS, Hemel Hempstead, UK) (Staff et al., 2014). The limit of quantification (LOQ) was 0.1 µg L^−1^; no samples were below this LOQ. The sampling device had variable residual Pb (blank devices contained 1.1 ± 0.6 µg L^−1^ of Pb). The spiked recoveries of blank saliva with known Pb concentrations added were taken through the method and were found to be 105 ± 21.1% at 2 µg L^−1^ and 102 ± 11.8% at 20 µg L^−1^ concentrations.

### Environmental samples

First draw tap water of the day (1 L) was collected in a HDPE bottle by study participants. The home tap water samples were collected in HDPE bottles and sent to Northumbrian Water Ltd., Howdon, UK, for analysis. Samples were acidified to 1% by the addition of 30 ml of 5 M Nitric Acid, placed in an oven at 80 ± 5 °C overnight and then stored at room temperature. Analysis for total Pb was undertaken using an in-house method HY-267; based on the ‘Blue Book’ method for Pb in potable water (Standing Committee of Analysts, 1976), but using inductively coupled plasma mass spectrometry. The laboratory is UKAS-accredited, and quality control is maintained by participation in Leap and Aquacheck proficiency schemes. The limit of detection for this method was 0.033 µg L^−1^. Methods and findings from the measurement of UAS soil Pb have been reported previously (Entwistle et al. 2019). In brief, researchers collected soil samples from around the roots of the crops (0–30 cm), as Pb uptake by crops was being investigated. Soil samples were analysed for their pseudo-total Pb concentration by Derwentside Environmental Testing Services (DETS), laboratories UK, using aqua regia digestion and analysis by ICP-OES.

### Questionnaires

All participants provided information on personal characteristics, including age, sex, smoking, alcohol consumption, occupations and hobbies that may involve some exposure to Pb, age of their home (as a proxy for the presence of lead paint in the home), whether they had Pb pipes for tap water, domestic cleaning habits and whether they kept cats or dogs as pets. Information on food preparation habits such as washing and peeling was collected. UAS gardener participants also provided information on frequency and duration of UAS visits, and habits like hand washing before eating on site, and washing and peeling of allotment produce. The food frequency questionnaire (FFQ) used was the IARC EPIC FFQ questionnaire (IARC, 1999) adapted to assess only fruit and vegetable consumption and to include the proportion of these that had been grown on UAS. Participants completed the questionnaires on paper, and information was uploaded into a Qualtrics survey database (Qualtrics, Provo, UT). The questionnaire is available on request from the corresponding author. The participant questionnaire, blood Pb, tap water and soil data were merged into one dataset for the analysis. Fruit and vegetable consumption rates were calculated as g food weight per kg body weight per day using average male (83.6 kg) and female (70.2 kg) UK body weights from the UK Office for National Statistics (BBC, 2010). Average adult portion sizes were obtained from various sources (MAFF 1993, NHS 2018), and average crop weight consumed per week (g) was calculated. The average portion size was multiplied by reported daily portion consumption for individual foods to give a median weight consumed per day for each crop type. These weights were summed per crop group to produce median daily consumption rates for each group. These consumption rates were divided by average UK body weights to give the g food weight consumed per kg body weight per day. The crops were summed according to crop groups set out in the Contaminated Land Exposure Assessment (CLEA) model (EA, 2009), the model used by UK regulatory bodies. Examples of the crops in each group are tuber: potatoes; root vegetables: carrots, onions, beetroot and rhubarb; green vegetables: cauliflower, lettuce, beans and asparagus; herbaceous fruit: strawberries, tomatoes, sweetcorn and courgettes; shrub fruit: raspberries, gooseberries and blueberries; and tree fruit: apples, cherries and grapes. The fraction eaten that was home-grown (HG) for each crop group was then estimated. The median weight consumed per day for each crop type for each participant was multiplied by the fraction reported by the participant as home-grown. The median UK adult portion sizes used for each crop are presented in Entwistle et al. (2019) along with the P75 portion size for reference and the source of the value. Several questionnaires were incomplete, missing only a single answer. Four saliva samples from three participants did not provide sufficient saliva sample for analysis, and three different participants did not provide sufficient blood sample for analysis. Where the number of data points missing from a variable was < 5%, multiple imputation was used to impute the missing data so as not to lose the participant’s data. An 8.45% missing proportion of alcohol units consumed was accepted, and multiple imputation was used as this was a variable of interest.

### Data analysis

Summary statistics and charts describing the demographics and characteristics of the participants and the environmental Pb concentrations were prepared. Geometric means and log_e_-transformed values were used when analysing saliva and blood Pb because the data were log-normally distributed. A Mann–Whitney test was used to compare BLL in UAS gardeners and controls, whilst the Kruskal–Wallis test was used to compare soil Pb levels between sites. A multiple linear regression model was developed in order to determine the predictors of log_e_ blood Pb concentration. Exposure effects were expressed as linear regression coefficients with p values and 95% confidence intervals. All statistical analyses were conducted using R version 3.4.1. Missing data were assumed to be missing at random and handled by multiple imputation method. Stepwise method was used for model selection. Predictors for BLL were initially determined separately for the UAS gardener and control groups to ensure that all significant variables for both groups were included in the final model. The regression model for the UAS gardeners included non-gardening- as well as gardening-related variables (potential BLL predictors) to determine UAS gardener variables (behaviours) significant for predicting BLL. The control model included only non-gardening variables.

## Results

We recruited 72 participants, 43 UAS gardeners from the three selected UAS and 29 controls. 60% of both UAS gardeners and controls were female. The majority of UAS gardeners were aged 41–59 years (60%), 33% were ≥ 60 years old, and 7% were ≤ 40 years old. The control group had a more even distribution of ages, 36%, 39% and 25% for the same age groups, respectively. Additional participant demographics are presented in Table 1.

**Table 1:** Counts, percentage of participants, blood lead concentration ranges and geometric means for selected participant demographics and characteristics

Participant/other characteristics		Gardeners		Controls		All		Blood lead (μg dL^−1^) [ZPP (µg g^−1^ Hb)]	
		N	%	N	%	N	%	Range	Geometric mean
Blood Pb level (μg dL^−1^)	All participants	–	–	–	–	70	100	0.6–4.1	1.49
	Gardeners	42	60	–	–	–	–	0.6 – 4.1	1.53
	Controls	–	–	28	40	–	–	0.7 – 2.9	1.43
ZPP level (µg g^−1^ Hb)	All participants	–	–	–	–	69	100	[2.0 – 8.4]	[3.29]
	Gardeners	41	60	–	–	–	–	[2.0 – 5.2]	[3.20]
	Controls	–	–	27	40	–	–	[2.5 – 8.4]	[3.44]
Age (years)	< 40	3	7	7	25	10	14	0.6–1.8	0.88
	40–59	25	60	10	36	35	50	0.6–3.7	1.66
	60 +	14	33	11	39	25	36	0.7–4.1	1.57
Gender	Female	26	62	17	61	43	61	0.6–3.7	1.48
	Male	16	38	11	39	27	39	0.7–4.1	1.51
Smoking	Never	24	57	15	54	39	56	0.6–2.9	1.44
	Former	16	38	12	43	28	40	0.7–4.1	1.52
	Current	2	5	1	4	3	4	1–2.7	1.89
Pb concentration in domestic tap water	Tertile 1 (0.01–0.10 µg L^−1^)	16	38	7	25	23	33	0.6–2.9	1.54
	Tertile 2 (0.11–0.50 µg L^−1^)	11	26	12	43	23	33	0.7–3.7	1.48
	Tertile 3 (0.63 – 12.0 µg L^−1^)	15	36	9	32	21	34	0.6–4.1	1.60
Average (mean) units of alcohol consumed per week	1–2	14	37	9	35	23	35	0.6–2.9	1.37
	3–4	15	39	9	35	24	37	0.6–4.1	1.42
	5–8	9	24	8	31	18	28	0.8–3.7	1.74
Hand to mouth behaviours (bite nails, etc.)	Yes	9	22	6	22	15	22	0.7–3.7	1.49
	No	32	78	21	78	53	78	0.6–4.1	1.49
Education	Up to O-levels/CSE/GCSE	9	21	3	11	12	17	0.6–4.1	1.45
	Apprenticeship/vocational	5	12	2	7	7	10	0.7–3.1	1.57
	Post-16 years and higher degree, etc.	28	67	23	82	51	73	0.6–3.7	1.49
Employment status	Working	23	55	16	57	39	56	0.6–3.7	1.42
	Other	19	45	12	43	31	44	0.7–4.1	1.58
Occupational or hobby exposure to Pb	No	30	71	20	71	50	71	0.6–4.1	1.37
	Yes	12	29	8	29	20	29	0.6–3.7	1.84
Pb pipes for domestic tap water	Yes	9	21	3	11	12	17	0.8–4.1	1.95
	No	13	31	6	21	19	27	0.7–2.9	1.53
	Don’t know	20	47	19	68	39	56	0.6–3.7	1.37

ZPP = Zinc protoporphyrin, Hb = haemoglobin

### Human biological data

**Fig. 1:**
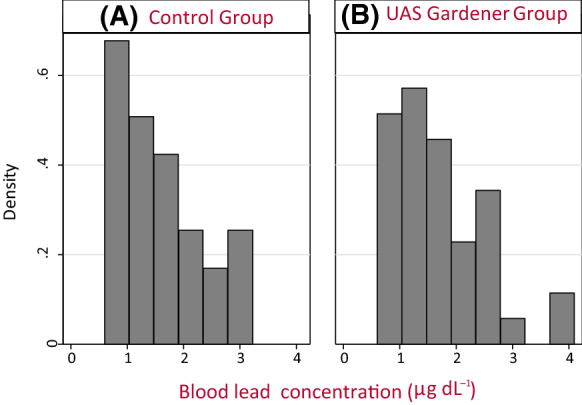
Distribution density of blood lead concentrations (µg dL^−1^) (number of counts for each age group) for control group (**A**) and urban agriculture site (UAS) gardeners (**B**)

The control group’s BLL range was 0.7–2.9 µg dL^−1^ with geometric mean 1.43 µg dL^−1^. The UAS gardeners’ BLLs ranged from 0.6 to 11.4 µg dL^−1^. An UAS gardener with a BLL of 11.4 µg dL^−1^ was removed from the data analyses as follow-up indicated that the raised level was linked to renovating leaded windows in the week prior to testing. The individual’s ZPP level was not elevated (at 2.5 µg g^−1^ Hb) and supported the self-reported claim of the exposure being an isolated recent occurrence. When this result was removed from the data set, the UAS gardeners’ BLL range was 0.6–4.1 µg dL^−1^ and the geometric mean was 1.53 µg dL^−1^ (see Table 1 and Fig. 1). There was no statistically significant difference between the BLL of the UAS gardeners and those of the non-gardening control group (p = 0.391). The range of saliva Pb concentrations (mean of the two saliva samples collected) was 1.1 to 4.5 µg L^−1^ with geometric mean 2.4 µg dL^−1^ for the control group, and the range was 1.3 to 7.9 µg L^−1^ with geometric mean 3.12 µg L^−1^ for the UAS gardeners. The difference in concentration between the first and second saliva samples ranged from 0.005 to 6.2 µg L^−1^ (24% to 376%) with median 1.2 µg L^−1^ (99%) (Supplementary Information, Table SI1).

### Environmental data

**Fig. 2:**
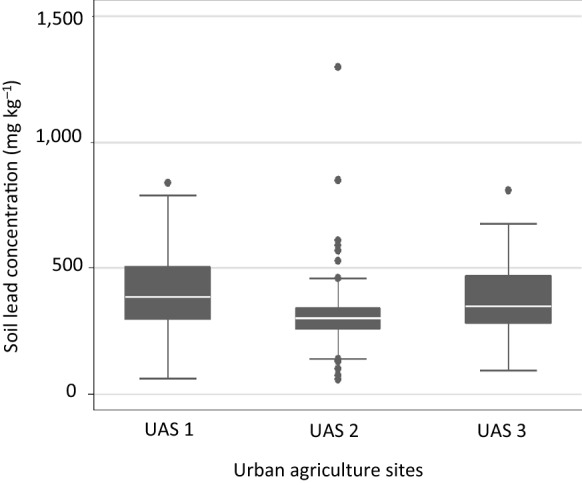
Box and whisker plots showing the median (white bar), first and third quartiles (lower and upper box boundary), maximum and minimum of soil lead concentrations (mg kg^-1^) in each of the study urban agriculture sites (UAS). Outlier concentrations ( ≥ 1.5 box lengths from the median) are shown with solid dots

Nearly 280 soil samples were collected from 31 UAS garden plots. Soil Pb concentrations for the three UAS had a geometric mean of 327 mg kg^−1^ and a 95^th^ percentile of 680 mg kg^−1^. A summary of soil Pb concentrations is presented in Fig. 2. Variability of soil Pb concentrations between the three UAS was not significant (*p* = 0.234); however, it was significant between individual gardens at the same site, potentially demonstrating the effect of different gardening practices both current and historic on Pb concentrations. Investigation of the drivers of this intra-site variability is beyond the scope of this current paper and as such is not considered further here. Home tap water Pb concentrations ranged from 0.06 to 12.0 µg L^−1^ (geometric mean 0.43 µg L^−1^). Where participants thought they had Pb pipes for domestic water (17% of participants), the geometric mean Pb concentration was 3.49 µg L^−1^. 55% of participants did not know whether they had Pb pipes in their home, and the geometric mean for their domestic tap water Pb was 0.24 µg L^−1^. 28% reported that they did not have Pb pipes or solder in their home, and their home tap water Pb concentrations had geometric mean 0.23 µg L^−1^. The highest measured Pb concentration in tap water (12.0 µg L^−1^, not flushed) was for a home where occupants were aware of their Pb pipes and flushed out standing water prior to the use of tap water for drinking or cooking. A summary of domestic tap water Pb concentrations is presented in Table 2, and the full data set is available as Supplementary Information Table SI2.

**Table 2:** Frequency of occurrence of Pb water pipes in the cohort and frequency of flushing of Pb water pipes (i.e. running the tap to flush out water that has spent time in the pipes, potentially containing dissolved Pb, before use of water for drinking or cooking) and resultant concentration range and geomean of Pb in domestic tap water

Participant has domestic lead water pipes or solder	% of Participants	Tap water Pb concentrations (µg L^-1^)		
		Minimum	Maximum	Geometric mean
Don't know	55 (n = 39)	0.014	3.8	0.24
No	28 (n = 20)	0.045	4.7	0.23
Yes	17 (n = 12)	0.5	12	3.49
Participants with domestic lead water pipes or solder and who flush/don’t flush their pipes				
Flush	8.5 (n = 6)	1.8	12	5.04
Don’t flush	8.5 (n = 6)	0.5	6.5	2.42

### Questionnaire data

Of all the participants, 29% reported some form of occupational or hobby exposure to Pb (Table 1). The most frequently reported exposures were from residential remodelling and construction (DIY) (n = 11) and fishing (n = 6). Other potential exposures were pottery making (n = 2), auto repair (n = 2), stained glass work (n = 2), furniture renovation (n = 1) and silversmithing (n = 1). People who grow their own fruit and vegetables are reported to be amongst the highest consumers of these crops (CL:AIRE, 2014). Geometric mean fruit and vegetable consumption rates for UAS gardeners and their non-gardening control neighbours are presented next to national UK data in Table 3. Although our UAS gardeners do appear to eat more fruit and vegetables than the control cohort, the differences were not statistically significant (Mann–Whitney test: green vegetables *p* = 0.61; herbaceous fruit *p* = 0.14; root vegetables *p* = 0.23; shrub fruit *p* = 0.13; tree fruit *p* = 0.55; tubers *p* = 0.09).

**Table 3:** Estimates of the amount of different fruit and vegetable groups eaten by adults in the Newcastle Allotments Biomonitoring Study (NABS) cohort, and UK wide survey data. Crop group consumption rates (in g food weight per kg bodyweight per day) for our UAS gardeners and controls (50th to 75th percentile [P] data) compared with findings from the UK National Diet and Nutrition Survey (NDNS, 2010/11; 50th to 90th percentile data), and % home-grown proportions in the diet of our UAS gardeners compared with data from the UK Expenditure and Food Survey (2004/5)

	Crop consumption rates (g fw kg^−1^ bw day^−1^)			Home-grown proportion (%)	
Crop group	NABS UAS gardeners P50–P75	NABS Controls P50–P75	UK NDNS Survey (2010/11) P50–P90	NABS UAS gardeners P50–P90	UK Expenditure and Food Survey Allotment gardeners (2004/5) ^a^
Green vegetables	2.5–3.7	2.0–3.0	1.26–2.36	35–69	33
Root vegetables	2.2–3.1	1.6–2.3	0.6–1.12	35–55	40
Tubers	2.4–3.3	1.7–2.3	1.18–2.35	30–100	13
Herbaceous fruit	2.5–3.7	2.0–3.1	0.69–1.29	21–73	40
Shrub fruit	0.7–1.1	0.3–0.5	0.09–0.18	54–99	60
Tree fruit	2.3–3.3	2.2–3.1	1.27–2.38	0–42	27

^a^as reported in EA, 2009c. Updated technical background to the CLEA model. Science Report – SC050021/SR3. ISBN: 978–1-84,432–856-7. Environment Agency

### Predictors of adult blood Pb levels

**Fig. 3:**
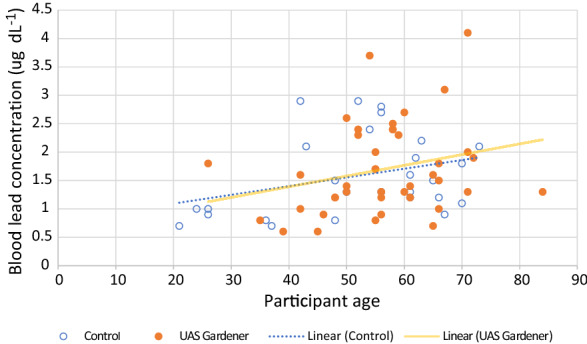
Scatter plot with linear trendlines to show the trend of increasing blood lead concentration with increasing participant age

Although our UAS gardener cohort had a slightly higher geometric mean BLL (1.53 µg dL^−1^) than the non-gardening cohort (1.43 µg dL^−1^), the difference was found to be non-significant by the multivariate linear regression model which included all collected potentially confounding Pb exposure data (p = 0.391). Categorised questionnaire responses, full sets of linear regression coefficients with standard errors, p values and 95% confidence intervals and excluded variables for the whole cohort and UAS gardener and control-only cohorts are provided in Supplementary Information Tables SI4–SI6. BLL predictors with significance *p* < 0.05 are discussed below and summarised in Supplementary Information Table SI7. Being older (*p* = 0.004) and being male (*p* = 0.004) were significant predictors of higher BLL for the cohort as a whole (including both UAS gardeners and non-UAS gardeners) (Fig. 3). For each year of age, BLL increased by 1.3%. We did not find home tap water Pb concentration to be a predictor of BLL for the whole cohort (*p* = 0.109). The number of years of gardening on UAS with high soil Pb was a significant predictor of higher BLL (*p* = 0.008), whereas the Pb concentration in the soil was not (p = 0.06). Dietary predictors of higher BLL for the whole cohort were eating more non-home-grown root vegetables, tubers and tree fruit. Eating more shrub fruit (either home-grown or non-home-grown) was also a predictor of higher BLL. Green vegetable consumption (either home-grown or non-home-grown) was identified as a significant predictor of lower BLL for the whole cohort. Although we included questions to investigate the impact on BLL of UAS gardener behaviours such as washing hands before eating when at the UAS, and peeling tubers and roots prior to eating, these variables were not significant for the regression model and excluded by stepwise model selection. The impact of frequency and thoroughness of washing crops before cooking or eating was also considered; however, the results were contradictory, possibly due to an unclear question format. Age of home residences was investigated as a potential BLL predictor (as a proxy for the presence of Pb paint); however, the majority of participants lived in pre-1930s homes (n = 60) with only five in home from the 1980s or later. When considering control participants only, additional predictors of higher BLL were participating in activities with potential Pb exposure, having pet cats or dogs, being a current smoker and having ‘fair’ self-reported health rather than good or very good. The UAS gardener group had different additional significant predictors of higher BLL: having a UAS in an urban rather than peri-urban location, having more years gardening on their current UAS, making shorter visits to the UAS, less frequent dusting at home, drinking more alcohol and going out to work. The UAS gardener group demonstrated that participants who had Pb domestic water pipes but did not flush them had higher BLL than participants with Pb domestic water pipes who did flush them, and those who did not have, or did not know if they had, Pb domestic water pipes. Eating more herbaceous fruit (either home-grown or non-home-grown) was another predictor of lower BLL for the UAS gardener cohort, as was eating home-grown tree fruit (Supplementary Information Table SI7).

**Table 4:** Comparison of Newcastle Allotments Biomonitoring Study (NABS) blood lead concentrations with previous studies. Note that the outlier due to occupational exposure has been excluded from all analyses

	Blood Pb µg dL^−1^ geometric mean (range) *n*			Blood Pb µg dL^−1^ geometric mean (P10-P95)				
Study country	This study, UK			Spain	France	USA	Korea	Sweden
Cohort name	NABS			BIOAMBIENT.ES^d^	ENNS^e^	NHANES^f^	KoNEHS^j^	Riksmaten^g^
Study date	2015			2009–10	2006–7	2015–16	2012–14	2010–11
Participant numbers	72			1880	2029	4988	4,000	273
Population	Gardeners	Controls	All		Cross section of adult population			
18–29 years F&M	1.8 *1*	0.9 (0.7–1.0) *4*	1.0 (0.7–1.8) *5*	1.9 (1.1 -4.6) *372*	1.9 (1.0–4.8) *604*	na	na	na
30–39 years F&M	0.7 (0.7–0.8) *2*	0.7 (0.6–0.8) *2*	0.7 (0.6–0.8) *4*	2.2 (1.3–4.8) *764*		na	na	na
40–49 years F&M	1.0 (0.6–1.6) *6*	2.1 (0.8–2.9) *4*	1.2 (0.6–2.9) *10*	2.7 (1.5–5.9) *466*	2.9 (1.5 -7.3) *988* ^a^	na	na	na
50–65 years F&M	1.8 (0.8–3.7) *17*	2.1 (1.3–2.9) *6*	1.9 (0.8–3.7) *23*	3.4 (1.8–7.2) *278*		na	na	na
60–74 years F&M	1.7 (0.7–4.1) *13*	1.5 (0.9–2.2) *11*	1.6 (0.7–4.1) *24*	na	3.9 (2.1—10.2) *437*	na	na	na
18–45 (F of CBA)	0.7 (0.6–1.0) *3*	1.0 (0.7–2.9) *4*	0.9 (0.6–2.1) *7*	1.8 (1.1–3.6) *700*	na	na	na	na
All M	1.7 (0.7–4.1) *16*	1.3 (0.7–2.9) *11*	1.5 (0.7–4.1) *16*	2.8 (1.6–6.4) *962*	3.0 (1.2—8.5) *758*	na	2.28 (1.73–4.53)^i^ *2766*	1.5^b^ (0.7–2.9)^c^ *128*
All F	1.5 (0.6–3.7) *24*	1.5 (0.7–2.9) *16*	1.5 (0.6–3.7) *40*	2.0 (1.1–4.5) *918*	2.2 (1.0—5.8) *1271*	na	1.66 (1.24–3.39)^i^ 3689	1.2^b^ (0.5–2.5)^c^ *145*
Total F&M	1.5 (0.8–2.61)^h^ *40*	1.4 (0.8–2.74)^h^ *27*	1.5 (0.8–2.7)^h^ *67*	2.4 (1.3–5.7) *1880*	2.6 (1.2 -7.3) *2029*	0.9 (2.9) *2610*	1.94 (1.43–4.09)^i^	1.3^b^ (0.6–2.9)^c^ *273*

F = female, M = male, CBA = childbearing age, na = not available, P10 = 10% confidence level, P95 = 95% confidence level *n* = number in cohort

^a^subjects aged 40–59 years

^b^median

^c^P5-P95

^d^Canas et al. (2014) BIOAMBIENT.ES = a nation-wide cross-sectional epidemiological study in Spain

^e^Falq et al. (2011) ENNS = Etude National Nutrition Santé (The French Nutrition and Health Survey)

^f^CDC (2018) NHANES = USA National Health and Nutrition Examination Survey adults >/= 20 years old, P95 only

^g^Bjermo et al. (2013) Raksmaten was a Swedish national survey investigating dietary habits among adults

^h^P10-P90

^i^P25-P95

^j^Choi et al. (2017) KoNEHS Korean National Environmental Health Survey

## Discussion

Our study found that UAS gardeners with raised Pb concentrations in their soil (95th percentile 680 mg kg^−1^) did not have significantly higher BLL than their non-gardening controls. Geometric BLL means for the UAS gardener and control groups were 1.53 and 1.43 µg dL^−1^, respectively. The blood samples were analysed in duplicate with three replicates each time and the mean reported. Typically, there was an RSD of 5% for the blood Pb measurements for the analysis based on the certified reference material data. The study steering group chose a health risk threshold for Pb in blood of 5 µg dL^−1^ for the study, in line with international guidance at the time, e.g. CDC (2012) and NSW Health (2016). All participants in our study had BLLs below the 5 µg dL^−1^ health risk threshold, except one discounted as temporary occupational exposure. The levels reported in both the UAS gardeners and the controls are higher than those reported for the same time period in the US National Health and Nutrition Examination Survey (NHANES) biomonitoring background levels study. Since the phasing out of leaded paint and petrol since the mid-1970s, many countries worldwide, including the USA and Australia, have seen a significant drop in national blood Pb levels (BLL) to well below 5 µg dL^−1^. The NHANES study reported a continued reduction in the geometric mean BLL in the adult population from 1.75 µg dL^−1^ in 1999–2000 to 1.23 µg dL^−1^ in 2009–2010 and then 0.92 µg dL^−1^ in 2015–2016 (CDC, 2018). Whilst there is no equivalent to NHANES in the UK, data from other European national studies indicate a geometric mean of 0.89 µg dL^−1^ in Germany in 2005 (Heitland & Köster, 2006), 2.4 µg dL^−1^ in Spain in 2009–2010 (Cañas et al., 2014), 2.57 µg dL^−1^ in France in 2006–2007 (Falq et al., 2011), and 1.34 µg dL^−1^ in Sweden in 2010–2011 (Bjermo et al., 2013) (see Table 4).

To the authors’ knowledge, this is the first study to investigate soil Pb with matched UAS gardener’s blood and saliva Pb concentration. The Pb levels in saliva samples were tenfold lower than the Pb in the blood samples, and this has been observed previously (Morton et al., 2014; Nriagu et al., 2006). We found that the saliva data did not correlate well with the blood Pb results; the first saliva sample showed no significant correlation, but the second saliva sample did show a weakly significant correlation (for 61 paired samples when Pb blood vs Pb ‘Saliva sample 2’ *p* = 0.0177). Duplicate saliva samples were collected within 15 min of each other, either side of the blood sample collection, to minimise variation between samples. Using saliva presents some problems in the collection and preparation of the sample: the flow and ion content of saliva can vary significantly throughout the day; whole saliva may contain other substances such as food debris, bacteria and epithelial cells; and hand-to-mouth behaviour prior to sample collection could cause sample contamination (Barbosa et al., 2006). There is also no widely agreed method to adjust for how dilute/concentrated the saliva collected is (analogous to creatinine correction for the analysis of urine). Environmental exposures to Pb such as those in this study are lower than those found in the occupational field, and as such Pb in a saliva sample is an analytical challenge in terms of reproducibility of the samples and the low levels being measured with Pb in saliva occurring at approximately a tenth of the level in blood. Lack of correlation with BLLs may be attributed to variations in saliva Pb occurring as a result of analytical quality control, as well as nutritional and hormonal status of the individual; variations in saliva ion content occurring throughout the day are associated with varying flow rate (Barbosa et al., 2005). It is not clear whether this variation between the duplicate samples is as a result of the timings of the sampling, it is possible that the first sample was a ‘washout sample’ and the second sample the ‘true sample’ or the fact that the second sample was a ‘better’ samples with practice or whether the sample device itself was responsible, there were also problems with the extraction method of the liquid and the sampling device had variable residual Pb. (Blank devices contained 1.1 ± 0.6 µg L^−1^ of Pb.) It is also likely that a correlation at such low blood Pb would be difficult to ascertain as observed in previous studies (Staff et al., 2014). Whilst the Pb levels were low in the sampling device itself, the extraction of the saliva sample was difficult and not reproducible and requires further refinement. It must also be considered that there may be biological processes involved that justify the lack of reproducibility. Whilst this warrants further investigation and shows some positive outcomes with sample 2 correlating better with blood Pb at levels < 5 µg dL^−1^, for this study the saliva results were not suitable biological media for Pb testing in this context (at such low concentrations) (Supplementary Information, Table SI1).

BLL predictors, indicated by our preferred regression model, were a complex mix of previous Pb exposure, anthropometrics, environment, current activities and diet. We found that higher Pb soil concentration in UAS was not a significant predictor of higher BLL for UAS gardeners (p = 0.06), but interestingly the greater number of years of UAS gardening was (p = 0.008), even after adjustment for UAS gardeners’ ages. Our findings suggest that UAS gardening can be source of Pb exposure but that the exposure is not high enough to cause a significant difference between UAS gardeners and controls in this study. Our reported BLLs (range 0.6–4.1 µg dL^−1^, geometric mean 1.49 µg dL^−1^for the whole cohort) accord well with a number of other larger similar studies, e.g. Feinberg et al. (2018) observed a geometric mean of 1.13 µg dL^−1^ for adults in New York in 2013–2014 (n = 1201), whilst Bocca et al. (2013) determined a geometric metric mean of 1.99 µg dL^−1^ in adults in Italy in 2008–2011 (n = 1423).

Lead has a long residence time in the human body with half-lives of 5–17 years in bone (Rabinowitz, 1991) and 28–36 days in blood (Griffin et al., 1975; Rabinowitz et al., 1976). It could be expected that participating in activities (current or historic) with potential Pb exposure would be a predictor of higher BLL. However, this predictor was only significant for the control group (p < 0.0005; Supplementary Information Table SI6). The ‘additional activities’ of the UAS gardeners that might expose them to Pb was not a significant predictor of BLL. This may be because the Pb exposure from these other activities was not discernible over and above the gardening-related Pb exposure. Our finding that BLLs increased with age (< 40 y 0.88 µg dL^−1^, 40–59 y 1.66 µg dL^−1^ and > 60 y 1.547 µg dL^−1^) is in keeping with previously reported studies of larger cohorts such as those in Spain (n = 1880) (Cañas et al., 2014), France (n = 2029) (Falq et al., 2011), Brazil (n = 374) (Takeda et al., 2017) and the Czech Republic (n = 1188) (Batáriová et al., 2006) (see Table 4). Older people have usually had more potential exposures to Pb through environmental exposure (such as Pb in petrol), hobbies or occupational exposure (such as fishing or renovation work in our cohort) (Vig & Hu, 2000). Lead is accumulated in the body throughout a lifetime and released very slowly. Its absorption depends on nutritional status, age and health of an individual as well as the route of exposure, particle size and chemical form. Absorbed Pb is mineralised in cortical and trabecular bone which have labile and inert components. Under normal healthy conditions, the inert bone component can store Pb for decades, but Pb mobilisation will take place during osteoporosis, lactation, pregnancy, menopause, hyperthyroidism, kidney disease, bone breaks and immobilisation for example. The smaller labile component readily transfers bone and blood Pb (ATSDR, 2010; Wani et al., 2015). For the control group only, self-reported poor health compared with good health was associated with higher BLL. Lead and health are intrinsically linked by both detrimental health effects of Pb exposure and release of Pb from bones during periods of physiological stress. The therapeutic effects of UAS gardening are acknowledged (Leake et al., 2009; Van Den Berg et al., 2010; Van Den Berg & Custers, 2011; Wakefield et al., 2007), and perhaps an indication of why the gardening cohort self-reported better health. Only 7% of participating UAS gardeners and 25% of controls were aged < 40 years. As such, most participants will have been exposed to higher environmental Pb levels in their lifetime, prior to the removal of Pb from petrol. A similar study comparing UAS gardeners and controls born after the ban of Pb in petrol is required to determine whether a lower baseline blood level would mean UAS gardeners with soils with raised Pb concentrations did then have significantly higher BLL than controls.

Our finding that male participants had marginally higher BLL (range 0.7–4.1, geometric mean 1.51 µg dL^−1^) than females (range 0.6–3.7, geometric mean 1.48 µg dL^−1^) is in keeping with previous studies of larger cohorts in Spain (Cañas et al., 2014), France (Falq et al., 2011), Korea (Choi et al. 2017) and Sweden (Bjermo et al., 2013) (see Table 4) that found males to have higher BLL than females. Although, at first glance, the difference between male and female geometric mean BLL appears negligible (Table 1), for our study the linear regression model (Supplementary Information Table SI4) found the difference to be significant, suggesting that the confounding exposure data collected strengthened this difference. Higher male BLL is attributed to three main factors: (1) higher Pb exposure in male lifestyles such as occupations, smoking and alcohol consumption, (2) the higher bone turner over in women during pregnancy, lactation and menopause results in reduced BLL after these periods and (3) Pb binds to red blood cells and men have a higher ratio of red blood cells in whole blood (Vahter et al., 2007). Working in employment with known Pb exposure was an exclusion factor for participants in the study, yet despite this we found that participants going out to work (rather than house husbands/wives or students) had higher BLLs (with geometric means 1.42 vs 1.37 µg dL^−1^, respectively). The association between employment and higher BLL suggests potential exposure from the wider urban environment, perhaps atmospheric Pb, increased soils track back to the home, or exposure during travel-to/for-work related transport. Having a UAS in an urban rather than peri-urban location was a predictor of higher BLL (p = 0.005), suggesting a link with either historic (e.g. aerial deposition from Pb in petrol) or current urban conditions. Resongles et al. (2021) reported Pb isotope composition in particles in London air to indicate historic leaded petrol to be a key source, the Pb being remobilised from urban soils. The control group only BLL predictor of keeping pet cats or dogs may relate to the animal tracking back outdoor soil into the home as well as track back from owners after dog walking.

Having Pb plumbing did not necessarily lead to higher BLL if care was taken to flush out sitting water before collecting water to drink or cook with. Those who recorded having Pb pipes exhibited a BLL of 1.95 µg dL^−1^ compared to 1.53 µg dL^−1^ in those who said they did not have Pb pipes. Whilst Pb pipes have long been known as a source of Pb in domestic water supply, this finding is somewhat surprising given UK water companies currently add orthophosphoric acid to water supplies to reduce dissolved Pb in water supplies (Jackson & Ellis, 2003) in order to comply with Water Supply (Water Quality) Regulations 2001 for England and Wales of a maximum of 10 µg L^−1^ Pb at consumers taps.

Higher Pb concentrations in UAS soil (95^th^ percentile of 680 mg kg^−1^) were not a key predictor (*p* < 0.06) for higher UAS gardener BLL. We found it was the number of years of UAS gardening and the behaviour relating to soil exposure pathways that were significant. We found those reporting shorter UAS visit length to have higher BLL. Although unexpected, this may indicate less time/considered need to change footwear and clothing so increased track back of soil into the home. Our findings regarding the importance of washing produce before eating were contradictory. Washing produce less often predicted a higher BLL for UAS gardeners, whereas never washing was not a significant predictor of higher BLL. Our finding that less frequent house dusting frequency was a predictor of higher BLL for UAS gardeners and the whole cohort, whilst vacuuming frequency was not a BLL predictor, may highlight the importance of smaller more frequently airborne particles as a Pb exposure route. Paustenbach et al. (1997) estimated that 50% of house dust originates from outside soils. Tu et al. (2020) found dust Pb to be more associated with track in from yard or garden soil. Park and Paik (2002) found a stronger correlation between BLL and respirable Pb concentration—that with particles size ≤ 1 µm, than with the more commonly measured airborne Pb concentration. Smaller particles make their way further into the respiratory tract. Several previous studies have previously found significant associations between Pb levels in house dust and BLL in children (Dixon et al., 2009; Levallois et al., 2014; Lioy et al., 1988.). The importance of particle size and inhalation may be the reason we found that less frequent house dusting (usually removing smaller particles) was a significant predictor of higher BLL for the whole cohort.

Higher alcohol consumption was a predictor of higher BLL for our UAS gardener only cohort (*p* = 0.002) as was being a current smoker (*p* = 0.05). Participants with reported average consumption of 5–8 units of alcohol consumed per week had BLL geometric mean 1.74 µg dL^−1^, whereas those reporting consumption of 1–2 units per week had BLL geometric mean 1.37 µg dL^−1^. Alcohol consumption has previously been linked to BLL due to the Pb present in alcoholic beverages, with Pb levels depending on the drink consumed; wine generally contains more Pb than beer, which in turn contains more than spirits (Weyermann & Brenner, 1997). The Pb is reported to come from the use of diatomaceous earth in drink filters used to improve clarity and lengthen shelf life (Redan et al., 2019). A difference in alcohol metabolism between sexes has also been reported (Weyermann & Brenner, 1997). Weyermann and Brenner (1997) also report a dose–response relationship between the number of cigarettes smoked and each day and BLL has, with increased contact with tobacco (filterless cigarettes, pipes and cigars), indicated to cause a greater BLL increase than filter-tipped cigarettes. Nail biters and cigarette smokers have been shown to have significantly higher BLL than their other work colleagues; often, simple increased hand-to-mouth contact causes such an increase (Gidlow, 2015).

We found that eating more green vegetables (either home-grown (p = 0.21) or non-home-grown (p = 0.001)) was significantly associated with lower BLL for the cohort as a whole. The UK Avon Longitudinal Study of Parents and Children (ALSPAC) (n = 4285) also reported a positive association between consumption of more green vegetables and lower BLL (Taylor et al., 2019). Our UAS gardener group also demonstrated significant associations between eating more herbaceous fruit, e.g. courgettes and tomatoes, either home-grown (p = 0.008) or non-home-grown (p = 0.034) and eating more home-grown tree fruit (p0.013), and lower BLLs. The protective effects of a diet with calcium, iron and vitamin C in reducing Pb uptake have been previously reported (Kordas, 2017; Thayer, 2018). Conversely, we found eating more root vegetables and shrub fruit (home-grown shrub fruit (p = 0.048), and non-home-grown (roots p = 0.002, shrub fruit p = 0.010)) and non-home-grown tubers (p = 0.009) to be associated with higher BLL for the whole cohort. We have previously published the Pb concentrations of fruit and vegetables collected for this study (Entwistle et al., 2019). We found root crops to have the highest uptake of Pb (range of means for root vegetable species analysed was 0.005 to 0.50 mg kg^−1^ fresh weight (fw), median 0.023 mg kg^−1^ fw) followed by shrub fruit (mean range 0.009 to 0.025 mg kg^−1^ fw, median 0.019 mg kg^−1^ fw), green vegetables (mean range 0.009 to 0.069 mg kg^−1^ fw, median 0.012 mg kg^−1^ fw), herbaceous fruit (mean range 0.002 to 0.023 mg kg^−1^ fw, median 0.006 mg kg^−1^ fw), then tubers (potatoes only, mean 0.006, median 0.002 mg kg^−1^ fw), with tree fruit showing the lowest uptake (apples only, mean 0.001, median 0.001 mg kg^−1^ fw). Our finding that greater root vegetable consumption was associated with higher BLL is in keeping with evidence that root vegetables are the crops that uptake the most Pb from soil (Chaney et al., 2010; Entwistle et al., 2019). Non-home-grown tree fruit was associated with higher BLL (whole cohort, *p* = 0.036), whereas home-grown tree fruit consumption was associated with lower BLL (UAS gardeners only, *p* = 0.013). Historically commercial orchards used lead arsenate (PbHAsO_4_) pesticide treatments (Codling, 2011; Schooley et al., 2008) which could potentially be taken up into crops (Hood, 2006; McBride, 2013). Our finding that consumption of more shrub fruit (e.g. raspberries, blueberries) is a predictor of higher BLL no matter whether the fruit is home-grown or not is also in keeping with our finding that shrub fruit demonstrated the second highest Pb concentrations of the UAS food groups tested. Some UAS and commercial shrub fruit, e.g. raspberries, may not be washed prior to sale or eating, and so it is feasible that atmospheric or windblown soil particles remain attached to the fruit and consumed by those eating them. Other considerations could be that UAS gardeners may not have always washed hands either prior to eating berries in the garden and in general people may be less thorough with washing of non-home-grown produce.

### Study limitations

Although our study design was robust in accounting for multiple confounders to BLL, the study was limited by its specific geographical location, the number and age of participants, and potentially the range of sources of UAS Pb contamination (e.g. no industrial Pb sources). The sample size was limited by the number of volunteers at the participating UAS with only 72 participants, 43 UAS gardeners and 29 controls. The study was focussed on UAS in Newcastle upon Tyne, to address a specific need identified by previous investigations. The study should be expanded across other geographic regions to determine if findings are also applicable there. Using survey data rather than monitoring actual events occurring may lead to inaccuracies. These limitations may preclude the possibility to draw definitive conclusions about the relationship between urban gardening on soils and the study outcome. Having only adult participants was limiting as the driver for UAS soil Pb guideline values is based on the risk to young children at UAS. Information regarding the presence of lead paint (and any recent renovations) in homes would have been a valuable addition to the multivariate linear regression model data set, as would concentrations of Pb in house dust and airborne particulate matter, and garden or backyard soil. A full dietary assessment rather than just fruit and vegetables would allow investigation of the protective effects of calcium intake, such as milk and cheese reported by Weyermann and Brenner (1997). Spices used for cooking have also been indicated as Pb exposure sources (Hore et al., 2019), ones which were not considered in our study. Our characterisation of Pb exposure from alcohol and smoking was limited by lack of detail on type of alcohol consumed or method and frequency of tobacco smoking. It is likely that a larger study would be required to fully account for the effects of these confounding variables in Pb exposure. In addition, we did not explore whether participants used a moistened dusting cloth, either with antistatic polish or with water to reduce resuspension of dust particles. This information is important when seeking to give advice on exposure reduction and should be included in future investigations.

### Implications

With an increasing focus on food security and sustainable production, and a growing awareness of the wider ecosystem services provided by greening our cities, regulators and planners need confidence in the ability of UAS to provide a safe food source as well as urban gardening as a healthy activity and valuable green space. This study has given some confidence to UK local authorities and UAS associations that urban gardens can be safe to use and can provide a valuable source of fresh fruits and vegetables, even where soil Pb levels are up to ten times above the UK’s recommended Pb screening level (Category 4 Screening Levels; CL:AIRE consortium, 2014). Without such evidence, closure and redevelopment of some UAS sites are a real concern. The study’s measurements of matched soil Pb concentrations and vegetable uptake in UAS environments (Entwistle et al., 2019) may be used in refining exposure assessment models. In addition to providing vital evidence on Pb exposure in Newcastle UAS, the study has placed a spotlight on non-UAS sources of Pb exposure, suggesting where resources may be focussed to target interventions for reducing urban Pb exposure. The apparent continuing risk from Pb plumbing in homes and the importance of appropriate PPE during occupational and DIY tasks involving potential Pb dusts warrant new/renewed public awareness campaigns. The apparent protective effects of a diet including plenty of green vegetables and herbaceous fruits in reducing Pb absorption may also be worth publicising.

In 2017, the Institute for Health Metrics and Evaluation (IHME) estimated that Pb exposure accounted for 1.06 million deaths and 24.4 million years of healthy life lost (disability-adjusted life years (DALYs)) worldwide due to long-term effects on health. The highest burden was in low- and middle-income countries (IHME, 2018). Guidance on acceptable BLL is driven by what is practical given historic exposures (such as Pb in petrol and paint) and emerging toxicological data. International BLL guidance (e.g. CDC 2012) commonly recommends a threshold for BLL of 5 µg dL^−1^. A low level of toxicological concern threshold of 3.5 has been proposed in the UK to be protective of renal toxicity and systolic high blood pressure in adults (DEFRA, 2014b). Two study participants were above this lower threshold: one male UAS gardener (age 71, 4.1 µg dL^−1^ BLL) and one female UAS gardener (age 54, 3.7 µg dL^−1^ BLL). Reducing Pb exposure to as low as reasonably practical is important for the health of those of all ages. Given the continued lowering of health exposure limits for Pb, in both adults and children, there is an increasing need to understand the apportionment and sources of bioavailable Pb. In this regard, the isotopic composition of blood Pb offers a potential way forward. Blood Pb values can be modelled using existing data for known anthropogenic sources (legacy leaded petrol, PM2.5–10 airborne particulates, coal ash, domestic and imported Pb ores used for the manufacture of in-house Pb pipes) (Hodgson et al., 2015; Shepherd et al., 2009, 2016). These will vary according to spatial and demographic factors but will provide an evidence-based isotopic framework for more informed public health intervention measures. Further research is necessary to include different types of Pb sources in UAS (such as industrial or sediment deposition during flood events) and different geographic areas, soil types, cultures and climates. Such work would be able to inform better safety regulation and guidance for the popular pastime of UAS gardening. Our findings also suggest that further investigation is warranted to determine if continued use of old orchards is a source of dietary Pb in non-home-grown produce, as well implications of the reuse of such sites as UAS.

## Conclusions

Urban gardening on soils with raised Pb concentrations is a global problem. This study found that growing and eating crops from the three UAS with high soil Pb concentrations that we investigated did not result in raised Pb body burden. The BLLs determined were within the level of concern stipulated by the study (5 µg dL^−1^). Similar findings may be anticipated for UAS gardeners on other UAS sites with similar soil types and Pb contamination sources, i.e. clay-rich soils with non-industrial and long weathered soil Pb. Use of  saliva samples for measuring lead body burden at the relatively low levels measured in this study requires further investigation. Significant predictors of higher BLL were increased age and being male. We found that eating more green and herbaceous vegetables predicted lower BLLs. Based on the findings of this study, to reduce urban Pb exposure we recommend that the following actions by both UAS gardeners and non-UAS gardeners alike could be effective as part of reducing exposure:

- Wear a protective mask when carrying out renovations on painted wood or leaded windows,
- Reduce track back of soils into home (via people and animals),
- Identify whether there is Pb present in the domestic water supply. Draw off a washing up bowl of water (more if the length of the pipe exceeds 40 m) before use for drinking or cooking if present (DWI, 1995),
- Eat a balanced diet with plenty of green vegetables and herbaceous fruit.

In addition to providing crucial evidence for the improved modelling of adult exposure to Pb in UAS scenarios, the study highlights several ‘everyday’ non-UAS Pb exposures. A new/renewed public health campaign on the sources of Pb still in our daily lives, alongside pragmatic ways to reduce exposure through raising the environmental health literacy of global citizens, would seem prudent. Our study highlights the critical role biomonitoring can play in exposure assessment, accounting for multiple routes of exposure. Including biomonitoring in screening programmes would provide vital evidence on effectiveness of current and future interventions aimed at improving public health. 

## Supplementary Information

Below is the link to the electronic supplementary material.

Supplementary file1 (DOCX 50 kb)

## Data Availability

Anonymised study data are available via the corresponding author.

## References

[CR1] Alaimo K, Packnett E, Miles RA, Kruger DJ (2008). Fruit and vegetable intake among urban community gardeners. Journal of Nutrition Education and Behavior.

[CR2] Alloway BJ (2004). Contamination of soils in domestic gardens and allotments: A brief overview. Land Contamination and Reclamation.

[CR3] Armstrong D (2000). A survey of community gardens in upstate New York: Implications for health promotion and community development. Heal Place.

[CR4] ATSDR. (2007). Toxicological profile for lead. U.S. Department of Health and Human Services, Public Health Service, Agency for toxic substances and disease registry 24049859

[CR5] ATSDR. (2010). Lead toxicity: What is the biological fate of lead? *Environ Heal Med Educ* 1–71

[CR7] Barbosa F, Tanus-Santos JE, Gerlach RF, Parsons PJ (2005). A critical review of biomarkers used for monitoring human exposure to lead: Advantages, limitations, and future needs. Environmental Health Perspectives.

[CR8] Barbosa F, Corrêa Rodrigues MH, Buzalaf MR, Krug FJ, Gerlach RF, Tanus-Santos JE (2006). Evaluation of the use of salivary lead levels as a surrogate of blood lead or plasma lead levels in lead exposed subjects. Archives of Toxicology.

[CR9] Batáriová A, Spěváčková V, Beneš B, Čejchanová M, Šmíd J, Černá M (2006). Blood and urine levels of Pb, Cd and Hg in the general population of the Czech Republic and proposed reference values. International Journal of Hygiene and Environmental Health.

[CR10] BGS. (2012). Normal background concentrations (NBCs) of contaminants in English soils: final project report. www.bgs.ac.uk/gbase/NBCDefraProject.html

[CR11] Bjermo H, Sand S, Nälsén C, Lundh T, Enghardt Barbieri H, Pearson M (2013). Lead, mercury, and cadmium in blood and their relation to diet among Swedish adults. Food and Chemical Toxicology.

[CR12] Bocca, B., Pino, A., & Alimonti, A. (2013). Metals as biomarkers of the environmental human exposure. *E3S Web of Conferences* 1, 260.10.1051/e3sconf/20130126004

[CR13] Bramwell, L., & Pless-Mulloli, T. (2008). Health risk assessment of urban agriculture sites using vegetable uptake and bioaccessibility data—an overview of 28 sites with a combined area of 48 hectares. ISEE Pasedena Abstract 874

[CR14] Buka, I., & Hervouet-Zeiber, C. (2019). Lead toxicity with a new focus: Addressing low-level lead exposure in Canadian children *Can Paediatr Soc Paediatr Environ Heal Sect.* Available: https://www.cps.ca/en/documents/position/lead-toxicity [Accessed 25 November 2019] 10.1093/pch/pxz080 PMC6587413 31239820

[CR15] Cañas AI, Cervantes-Amat M, Esteban M, Ruiz-Moraga M, Perez-Gomez B, Mayor J, ES B, Castaño A (2014). Blood lead levels in a representative sample of the Spanish adult population: The BIOAMBIENT. ES project. International Journal of Hygiene and Environmental Health.

[CR16] CDC. (1997). Centres for disease control prevention capillary blood sampling protocol

[CR17] CDC. (2018). *Fourth national report on human exposure to environmental chemicals: Updated Tables*, March 2018, Volume One

[CR18] CDC. (2019). Childhood lead poisoning prevention program. Centers Dis Control Prev. Available: www.cdc.gov/nceh/lead/

[CR19] Chaney, R. L., Codling, E. E., Scheckel, K. G., & Zia, M. (2010). Pb in carrots grown on Pb-rich soils is mostly within the xylem. *Am Soc Agron Annu Meet Long Beach, CA, ASA, Madison, WI* Abstract 60451

[CR20] CL:AIRE. (2014). SP1010—*Development of category 4 Screening levels for assessment of land affected by contamination*

[CR21] Clark HF, Hausladen DM, Brabander DJ (2008). Urban gardens: Lead exposure, recontamination mechanisms, and implications for remediation design. Environmental Research.

[CR22] Codling, E. (2011). *Environmental impact and remediation of residual lead and arsenic pesticides in soil*

[CR23] Costa de Almeida GR, Umbelino de Freitas C, Barbosa F, Tanus-Santos JE, Gerlach RF (2009). Lead in saliva from lead-exposed and unexposed children. Science of the Total Environment.

[CR24] Da WJ, Ren HM, Liu JS, Yu JB, Zhang XL (2006). Distribution of lead in urban soil and its potential risk in Shenyang City, China. Chinese Geogr Sci.

[CR25] Datko-Williams L, Wilkie A, Richmond-Bryant J (2014). Analysis of US soil lead (Pb) studies from 1970 to 2012. Science of the Total Environment.

[CR26] DEFRA/EA. (2002). *Soil guideline values for lead contamination*. ISBN 1 857 05736 8

[CR27] DEFRA. (2014b). SP1010: *Development of category 4 Screening levels for assessment of land affected by contamination—Policy companion document*

[CR28] DEFRA. (2014a). SP1010: *Appendix H provisional C4SLS for lead*

[CR29] Dixon SL, Gaitens JM, Jacobs DE, Strauss W, Nagaraja J, Pivetz T, Wilson JW, Ashley PJ (2009). Exposure of US children to residential dust lead, 1999–2004: II. The contribution of lead-contaminated dust to children’s blood lead levels. Environmental Health Perspectives.

[CR30] DWI, Department of the Environment/Welsh Office Drinking Water Inspectorate. (1995). Lead in Drinking Water - Have You Got Lead Pipes? 1995

[CR31] EA. (2009). CLEA Software (Version 1.05) *Handbook better regulation science programme science report: SC050021/SR4*. Environment Agency, UK

[CR32] Ekong EB, Jaar BG, Weaver VM (2006). Lead-related nephrotoxicity: A review of the epidemiologic evidence. Kidney International.

[CR33] Entwistle JA, Amaibi PM, Dean JR, Deary ME, Medock D, Morton J (2019). An apple a day? Assessing gardeners’ lead exposure in urban agriculture sites to improve the derivation of soil assessment criteria. Environment International.

[CR35] Falq G, Zeghnoun A, Pascal M, Vernay M, Le Strat Y, Garnier R, Olichon D, Bretin P, Castetbon K, Fréry N (2011). Blood lead levels in the adult population living in France he French Nutrition and Health Survey (ENNS 2006–2007). Environment international.

[CR36] Feinberg A, McKelvey W, Hore P, Kanchi R, Parsons PJ, Palmer CD (2018). Declines in adult blood lead levels in New York City compared with the United States, 2004–2014. Environmental Research.

[CR37] Filippelli GM, Laidlaw MAS (2010). The elephant in the playground: Confronting lead-contaminated soils as an important source of lead burdens to urban populations. Perspectives in Biology and Medicine.

[CR38] Gidlow DA (2015). Lead toxicity. Occup Med (Chic Ill).

[CR39] Gomaa A, Hu H, Bellinger D, Schwartz J, Tsaih SW, Gonzalez-Cossio T (2002). Maternal bone lead as an independent risk factor for fetal neurotoxicity: A prospective study. Pediatrics.

[CR40] Griffin T, Coulston F, Wills H (1975). Biological and clinical effects of continuous exposure to airborne particulate lead. *Arh Hig Toksikol.* (Yugoslavian). Arh Hig Toksikol.

[CR41] NSW Health. (2016). *Elevated Blood Lead Levels-Response Protocol for NSW Public Health Units*

[CR42] Heitland P, Köster HD (2006). Biomonitoring of 37 trace elements in blood samples from inhabitants of northern Germany by ICP-MS. Journal of Trace Elements in Medicine and Biology.

[CR43] Hodgson S, Manmee C, Dirks W, Shepherd TJ, Pless-Mulloli T (2015). Determinants of childhood lead exposure in the postleaded petrol era: The Tooth Fairy cohort from Newcastle upon Tyne. Journal of Exposure Science and Environmental Epidemiology.

[CR44] Hood E (2006). The apple bites back: Claiming old orchards for residential development. Environmental Health Perspectives.

[CR45] Hore P, Alex-Oni K, Sedlar S, Nagin D (2019). A spoonful of lead: A 10-year look at spices as a potential source of lead exposure. Journal of Public Health Management and Practice.

[CR46] IARC. (1999). Dietary exposure—EPIC. https://epic.iarc.fr/about/dietaryexposure.php

[CR47] IHME. (2018). *Findings from the Global Burden of Disease Study 2017*

[CR48] Jackson P., Ellis J. (2003). *Demonstration of optimisation of plumbosolvency treatment and control measures final report to the drinking water inspectorate*

[CR49] Koh D, Ng V, Chua LH, Yang Y, Ong HY, Chia SE (2003). Can salivary lead be used for biological monitoring of lead exposed individuals?. Occupational and Environmental Medicine.

[CR50] Kordas K (2017). The “Lead Diet”: Can dietary approaches prevent or treat lead exposure*?*. Journal of Pediatrics.

[CR51] Lanphear BP, Hornung R, Khoury J, Yolton K, Baghurst P, Bellinger DC (2005). Low-level environmental lead exposure and children’s intellectual function: An international pooled analysis. Environmental Health Perspectives.

[CR52] Lanphear BP, Rauch S, Auinger P, Allen RW, Hornung RW (2018). Low-level lead exposure and mortality in US adults: A population-based cohort study. Lancet.

[CR53] Latimer JC, Van Halen D, Speer J, Krull S, Weaver P, Pettit J (2016). Soil lead testing at a high spatial resolution in an urban community garden: A case study in relic lead in Terre Haute, Indiana. Journal of Environmental Health.

[CR54] Leake JR, Adam-Bradford A, Rigby JE (2009). Health benefits of “grow your own” food in urban areas: Implications for contaminated land risk assessment and risk management?. Environmental Health.

[CR55] Levallois P, St-Laurent J, Gauvin D, Courteau M, Prévost M, Campagna C, Lemieux F, Nour S, D'amour M, Rasmussen PE (2014). The impact of drinking water, indoor dust and paint on blood lead levels of children aged 1–5 years in Montréal (Québec, Canada). Journal of Exposure Science and Environmental Epidemiology.

[CR56] Lioy PJ, Yiin LM, Adgate J, Weisel C, Rhoads GG (1998). The effectiveness of a home cleaning intervention strategy in reducing potential dust and lead exposures. J Expo Anal Environ Epidemiol.

[CR57] Litt JS, Soobader MJ, Turbin MS, Hale JW, Buchenau M, Marshall JA (2011). The influence of social involvement, neighborhood aesthetics, and community garden participation on fruit and vegetable consumption. American Journal of Public Health.

[CR58] Martin CJ, Werntz CL, Ducatman AM (2004). The interpretation of zinc protoporphyrin changes in lead intoxication: A case report and review of the literature. Occup Med (chic Ill).

[CR59] McBride MB (2013). Arsenic and lead uptake by vegetable crops grown on historically contaminated orchard soils. Applied and Environmental Soil Science.

[CR61] Meharg AA (2016). Perspective: City farming needs monitoring. Nature.

[CR62] Mielke HW, Gonzales CR, Powell ET, Mielke PW (2016). Spatiotemporal dynamic transformations of soil lead and children’s blood lead ten years after Hurricane Katrina: New grounds for primary prevention. Environment International.

[CR63] Mitchell RG, Spliethoff HM, Ribaudo LN, Lopp DM, Shayler HA, Marquez-Bravo LG (2014). Lead (Pb) and other metals in New York City community garden soils: Factors influencing contaminant distributions. Environmental Pollution.

[CR64] Morton J, Leese E, Harding A-H, Jones K, Sepai O (2014). Saliva as a matrix for biomonitoring occupational and environmental exposure to lead. Biomonitoring.

[CR65] Navas-Acien A, Guallar E, Silbergeld EK, Rothenberg SJ (2007). Lead exposure and cardiovascular disease—A systematic review. Environmental Health Perspectives.

[CR66] BBC News (2010). Office for National statistics reveal Britain’s “Mr and Mrs Average.” https://www.bbc.co.uk/news/uk-11534042 Accessed 28/3/20

[CR67] NHMRC (2016). *Managing individual exposure to lead in Australia—A guide for health practitioners*. National Health Medical Research Council

[CR68] Nriagu J, Burt B, Linder A, Ismail A, Sohn W (2006). Lead levels in blood and saliva in a low-income population of Detroit, Michigan. International Journal of Hygiene and Environmental Health.

[CR69] NTP. 2012. US National toxicology program monograph *Health Effects of Low-Level Lead* 23964424

[CR70] ONS (2016). Office for National Statistics, National Records of Scotland, Northern Ireland Statistics and Research Agency, 2011 Census aggregate data (Edition June). UK Data Serv. Available.

[CR71] Park DU, Paik NW (2002). Effect on blood lead of airborne lead particles characterized by size. Annals of Occupational Hygiene.

[CR72] Paustenbach DJ, Finley BL, Long TF (1997). The critical role of house dust in understanding the hazards posed by contaminated soils. International Journal of Toxicology.

[CR73] PHE. 2014. Public Health England; Guidance, *Lead: health effects, incident management and toxicology*

[CR74] Pless-Mulloli T, Air V, Vizard C, Singleton I, Rimmer DL, Hartley P (2004). The legacy of historic land-use in allotment gardens in industrial urban settings: Walker Road allotment in Newcastle upon Tyne, UK. Land Contamination and Reclamation.

[CR75] Pless-Mulloli T, Paepke O, Schilling B. 2001. *PCDD/PCDF and heavy metals in soil and egg samples taken from Newcastle allotments: Assessment of the role of the byker incinerator. Full technical report*

[CR76] Rabinowitz MB (1991). Toxicokinetics of bone lead. Environmental Health Perspectives.

[CR77] Rabinowitz MB, Wetherill GW, Kopple JD (1976). Kinetic analysis of lead metabolism in healthy humans. The Journal of Clinical Investigation.

[CR78] Redan BW, Jablonski JE, Halverson C, Jaganathan J, Mabud MA, Jackson LS (2019). Factors affecting transfer of the heavy metals arsenic, lead, and cadmium from diatomaceous-earth filter aids to alcoholic beverages during laboratory-scale filtration. Journal of Agricultural and Food Chemistry.

[CR79] Rouillon M, Harvey PJ, Kristensen LJ, George SG, Taylor MP (2017). VegeSafe: A community science program measuring soil-metal contamination, evaluating risk and providing advice for safe gardening. Environmental Pollution.

[CR80] Rust SW, Kumar P, Burgoon DA, Niemuth NA, Schultz BD (1999). Influence of bone-lead stores on the observed effectiveness of lead hazard intervention. Environmental Research.

[CR81] Schooley T, Weaver M, Mullins D, Eick M (2009). the history of lead arsenate use in apple production: Comparison of its impact in Virginia with other states. Journal of Pesticide Safety Education.

[CR82] Shepherd TJ, Chenery SR, Pashley V, Lord RA, Ander LE, Breward N, Hobbs SF, Horstwood M, Klinck BA, Worrall F (2009). Regional lead isotope study of a polluted river catchment: River Wear, Northern England, UK. Science of the Total Environment.

[CR83] Shepherd TJ, Dirks W, Roberts NMW, Patel JG, Hodgson S, Pless-Mulloli T, Walton P, Parrish RR (2016). Tracing fetal and childhood exposure to lead using isotope analysis of deciduous teeth. Environmental Research.

[CR85] Staff JF, Harding AH, Morton J, Jones K, Guice EA, McCormick T (2014). Investigation of saliva as an alternative matrix to blood for the biological monitoring of inorganic lead. Toxicology Letters.

[CR86] Standing Committee of Analysts (1976). Lead in potable waters by atomic absorption spectrophotometry . Methods for the examination of waters and associated materials. HMO

[CR87] Takeda SHK, Kuno R, Barbosa F, Gouveia N (2017). Trace element levels in blood and associated factors in adults living in the metropolitan area of Sao Paulo, Brazil. Journal of Trace Elements in Medicine and Biology.

[CR88] Taylor CM, Doerner R, Northstone K, Kordas K (2019). Dietary patterns are not consistently associated with variability in blood lead concentrations in pregnant British women. Journal of Nutrition.

[CR89] Taylor MP, Isley CF, Fry KL, Liu X, Gillings MM, Rouillon M, Soltani NS, Gore DB, Filippelli GM (2021). A citizen science approach to identifying trace metal contamination risks in urban gardens. Environment International.

[CR90] Téllez-Rojo MM, Hernández-Avila M, Lamadrid-Figueroa H, Smith D, Hernández-Cadena L, Mercado A (2004). Impact of bone lead and bone resorption on plasma and whole blood lead levels during pregnancy. American Journal of Epidemiology.

[CR91] Thayer B. 2018. How to fight lead exposure with nutrition. eat eight academy of nutrition and dietetics, www.eatright.org/health/wellness/preventing-illness/how-to-fight-lead-exposure-with-nutrition accessed 28/3/20

[CR92] Tu JW, Fuller W, Feldpausch AM, Van Landingham C, Schoof RA (2020). Objective ranges of soil-to-dust transfer coefficients for lead-impacted sites. Environmental Research.

[CR93] Vahter M, Åkesson A, Liden C, Ceccatelli S, Berglund M (2007). Gender differences in the disposition and toxicity of metals. Environmental research.

[CR94] Van Den Berg AE, Custers MHG (2011). Gardening promotes neuroendocrine and affective restoration from stress. Journal of Health Psychology.

[CR95] Van Den Berg AE, Van Winsum-Westra M, De Vries S, Van Dillen SM (2010). Allotment gardening and health: A comparative survey among allotment gardeners and their neighbors without an allotment. Environmental Health A.

[CR96] Vig EK, Hu H (2000). Lead toxicity in older adults. Journal of the American Geriatrics Society.

[CR97] Vupputuri S, He J, Muntner P, Bazzano LA, Whelton PK, Batuman V (2003). Blood lead level is associated with elevated blood pressure in blacks. Hypertension.

[CR98] Wakefield S, Yeudall F, Taron C, Reynolds J, Skinner A (2007). Growing urban health: Community gardening in South-East Toronto. Health Promotion International.

[CR99] Wani AL, Ara A, Usmani JA (2015). Lead toxicity: A review. Interdisciplinary Toxicology.

[CR100] Weyermann M, Brenner H (1997). Alcohol consumption and smoking habits as determinants of blood lead levels in a national population sample from Germany. Archives of Environmental Health: An International Journal.

[CR101] WHO/JEFCA. 2011. WHO Technical report series 960 Evaluation of certain food additives and contaminants 22519244

