# Supplementary material for: Determinants of blood and saliva lead concentrations in adult gardeners on urban agricultural sites
Source: Environ Geochem Health. 2021 Oct 7;44(10):3493–513. doi: 10.1007/s10653-021-01095-7 (PMC9522656; doi:10.1007/s10653-021-01095-7)
Supplement: Supplementary file 1 — Supplementary file1 (DOCX 50 kb) [file 10653_2021_1095_MOESM1_ESM.docx]

**Determinants of lead body burden for adult gardeners on urban agricultural sites.**

**Supplementary Infomation**

**Lindsay Bramwell*^a1^, Jackie Morton^b^, Anne-Helen Harding^b^, Nan Lin^c^ and Jane Entwistle^c^**

*corresponding author

^a^Institute of Health and Society, Newcastle University, Newcastle upon Tyne, Tyne and Wear, NE4 5TG, UK

^1^Present address: Department of Geography and Environmental Sciences, Northumbria University, Ellison Building, Newcastle Upon Tyne, Tyne and Wear, NE1 8ST, UK

email: [lindsay.bramwell@northumbria.ac.uk](mailto:lindsay.bramwell@northumbria.ac.uk)

^b^Health and Safety Executive, Buxton, Derbyshire, SK17 9JN, UK

^c^Department of Mathematics, Physics and Electrical Engineering, Northumbria University, Ellison Building, Newcastle Upon Tyne, Tyne and Wear, NE1 8ST, UK

^d^Department of Geography and Environmental Sciences, Northumbria University, Ellison Building, Newcastle Upon Tyne, Tyne and Wear, NE1 8ST, UK

**Table SI1. Concentrations of Pb in first (Saliva A) and second (Saliva B) saliva concentrations for study participants.**

| **saliva A** | **saliva B** |  | **saliva A** | **saliva B** |
| --- | --- | --- | --- | --- |
| **µg/L** | **µg/L** |  | **µg/L** | **µg/L** |
| 2.983 | 1.574 |  | 1.557 | 1.079 |
| 1.959 | 1.422 |  | 4.287 | 1.815 |
| 0.779 | 1.977 |  | 3.264 | 7.051 |
| 5.289 | 3.343 |  | 4.59 | 3.619 |
| 2.353 | 6.523 |  | is | 3.489 |
| 3.061 | 0.822 |  | 1.793 | 1.336 |
| 3.495 | 3.125 |  | 2.166 | 5.582 |
| 1.943 | 2.842 |  | 2.498 | 3.316 |
| 7.044 | 3.782 |  | is | 2.697 |
| 5.273 | 3.542 |  | 2.544 | 1.592 |
| 2.47 | 4.241 |  | 3.271 | is |
| 3.151 | 3.511 |  | 2.678 | 3.014 |
| 4.792 | 2.515 |  | 3.106 | 2.734 |
| 9.608 | 4.183 |  | 1.274 | is |
| 1.454 | 1.493 |  | 1.812 | 2.19 |
| 2.057 | 3.395 |  | 4.791 | 2.936 |
| 3.942 | 2.509 |  | 2.117 | 2.064 |
| 1.603 | 2.706 |  | 0.979 | 1.18 |
| 4.773 | 2.731 |  | 9.299 | 6.514 |
| 2.138 | 1.139 |  | 1.677 | 1.08 |
| 1.894 | 1.981 |  | 2.092 | 4.073 |
| 2.857 | 3.485 |  | 1.571 | 0.609 |
| 2.699 | 1.83 |  | 2.278 | 1.38 |
| 1.672 | 3.515 |  | 5.422 | 3.272 |
| 1.933 | 3.638 |  | 7.177 | 1.756 |
| 1.152 | 2.674 |  | 2.281 | 1.433 |
| 1.777 | 3.039 |  | 4.135 | 4.703 |
| 4.106 | 2.979 |  | 3.528 | 8.987 |
| 2.105 | 6.803 |  | 4.362 | 2.369 |
| 3.783 | 3.33 |  | 6.176 | 2.383 |
| 2.237 | 8.403 |  | 3.12 | 3.154 |
| 0.895 | 1.546 |  | 1.412 | 3.617 |
| 1.171 | is |  | 3.349 | 3.67 |
| 2.963 | 2.958 |  | 3.584 | 3.708 |
| 2.52 | 3.677 |  | is | 3.719 |

is – insufficient sample

**Table SI2. Tap water Pb concentrations for study participants**

| **Tap water Pb** |  | **Tap water Pb** |
| --- | --- | --- |
| **µg/L** |  | **µg/L** |
| 3.8 |  | 0.41 |
| 0.049 |  | 0.22 |
| 6.5 |  | 4.2 |
| 0.32 |  | 0.45 |
| 0.10 |  | 0.41 |
| 0.12 |  | 0.16 |
| 0.35 |  | 2.0 |
| 0.084 |  | 0.11 |
| 0.21 |  | 0.13 |
| 0.063 |  | 0.13 |
| 0.045 |  | 0.16 |
| 0.084 |  | 3.2 |
| 0.50 |  | 0.062 |
| 0.35 |  | 0.06 |
| 0.051 |  | 12 |
| 2.1 |  | 4.7 |
| 0.034 |  | 12 |
| 1.8 |  | 0.099 |
| 0.021 |  | 0.069 |
| 0.1 |  | 0.1 |
| <0.014 |  | 1.1 |
| 0.15 |  | 3.3 |
| 2.9 |  | 0.14 |
| 0.10 |  | 0.4 |
| 0.63 |  | 2.3 |
| 1.1 |  | 0.40 |
| 0.061 |  | 2.3 |
| 0.077 |  | 0.12 |
| 0.055 |  | 0.099 |
| 1.3 |  | 0.063 |
| 0.18 |  | 0.062 |
| 0.42 |  | 4.6 |
| 3.2 |  | 0.29 |
| 4.2 |  | 1.8 |
| 4.7 |  | 2.4 |
| 3.1 |  |  |

**Table SI3. Questionnaire response categories and counts included in the multiple regression analysis**

| **Question** | **Responses** | **Number of people: number of controls, number of gardeners)** |
| --- | --- | --- |
| Are you male or female? | Female  Male | 43: control = 17, gardener = 26  28: control = 11, gardener = 17 |
| How would you rate your general health? | Bad or Very Bad  Fair  Good or Very good | 0  15: controls = 6, gardener = 9  56: controls = 22, gardener = 34 |
| Do you smoke or did you previously smoke? | Current  Previous  Never | 3: controls = 1, gardener = 2  29: controls = 12, gardener = 17  39: controls = 15, gardener = 24 |
| Do you bite your nails or have a similar hand to mouth habit? | Yes  No | 16: controls = 6, gardener = 10  55: controls = 22, gardener = 33 |
| How old is your home? | 1980s or later  1960/70s  1930s and earlier | 5  6  60 |
| Do you have pet cats or dogs in your home? | No  yes | 39: controls = 13, gardener = 26  32: controls = 15, gardener = 17 |
| Do you have lead plumbing at home and if so do you flush through water before taking water for drinking or cooking? | Lead pipe = Yes and run pipe = No  Lead pipe = Yes and run pip = Yes  Lead pipe = No  Lead pipe = Don’t know | 6:controls = 1, gardeners = 5  6:controls = 2, gardeners = 4  20:controls = 6, gardeners = 14  39:controls = 19, gardeners = 20 |
| How frequently do you vacuum or sweep your home? | Every day  5-6 times a week  2-4 times a week  Once a week  1-3 times a month  <once a month | 3  22  30  10  2  4 |
| How frequently do you dust your home? | 5-6 times a week  2-4 times a week  Once a week  1-3 times a month  <once a month | 14: controls = 2, gardener = 12  17: controls = 7, gardener = 10  15: controls = 6, gardener = 9  1: controls = 0, gardener = 1  24: controls = 13, gardener = 11 |
| What is your current employment status? | Housewife/husband or Unemployed  Student or working | 26: controls = 10, gardener = 16  45: controls = 18, gardener = 27 |
| Previous or current activities with potential lead exposure | Non  Some or lots | 50: control = 20, gardener = 30  21: control = 8, gardener = 13 |
| Do you have a UAS? | Control  Gardener | 28  43 |
| Is your UAS in a rural or urban location? | Peri Urban (site 1)  Urban (site 2 and 3) | 13  30 |
| Visit length at UAS | <= 2 hours and other  2-4 hours  4-6 hours | 16  19  8 |
| Visit frequency at UAS | 2-4 times a week  5-6 times a week  Every day  once a week | 19  8  1  14 |
| Bonfires on UAS | none or don’t know  a few  lots | 7  27  8 |
| Old window frames on UAS | none  a few | 22  20 |
| Pylon within 500 m of UAS site | YES (site 1)  No (other sites) | 13  30 |
| UAS near a major road | YES (site 3)  No (other sites) | 15  28 |
| Washing fruit and vegetables before eating | usually  sometimes  never | 4  17  21 |

**Questions not included in the regression analysis**

Ethnicity was not included as most participants were white British. Educational level due to wide diversity and issues with classification. Numbers of residents in homes. Lead paint homes – covers same information as home age. Pylon proximity to site. These were not considered key BLL predictors so were left out to allow greater clarity to those predictors of greater importance to the investigation.

**Table SI4. Whole Cohort: Results from the linear regression model showing predictors of log blood lead for the whole cohort using stepwise model selection.**

| **Predictors/Explanatory variables** | **p-value** | **Co-efficient** | **Standard error** | **95% confidence interval** | |
| --- | --- | --- | --- | --- | --- |
| Gardener (compared to control) | 0.391 | 0.168 | 0.194 | -0.223 | 0.559 |
| *Age (years) | 0.004 | 0.013 | 0.004 | 0.004 | 0.021 |
| *Gender: males compared to females | 0.004 | 0.340 | 0.111 | 0.116 | 0.564 |
| General health: bad compared to very good | 0.159 | 0.171 | 0.119 | -0.070 | 0.411 |
| Alcohol consumption (unit) | 0.197 | 0.031 | 0.024 | -0.017 | 0.079 |
| Tap water Pb concentrations (µg L^-1^) | 0.109 | 0.042 | 0.026 | -0.010 | 0.093 |
| Lead pipe: run water compared to not run water | 0.146 | -0.331 | 0.224 | -0.783 | 0.120 |
| No lead pipe compared to having lead pipe and not run water | 0.401 | -0.345 | 0.408 | -1.166 | 0.475 |
| Don’t know’ compared to having lead pipe and not run water | 0.816 | -0.093 | 0.399 | -0.897 | 0.710 |
| House dusted  (frequency from 5-6 times a week to less than once a month) | 0.051 | -0.077 | 0.039 | -0.155 | 0.000 |
| **Non-homegrown green vegetable consumption (g kg^-1^ bw d^-1^) | 0.001 | -0.158 | 0.043 | -0.244 | -0.072 |
| **Homegrown green vegetable consumption (g kg^-1^ bw d^-1^) | 0.021 | -0.212 | 0.088 | -0.390 | -0.034 |
| *Non-homegrown root vegetable consumption (g kg^-1^ bw d^-1^) | 0.002 | 0.165 | 0.050 | 0.064 | 0.265 |
| *Non-homegrown tuber consumption (g kg^-1^ bw d^-1^) | 0.009 | 0.056 | 0.021 | 0.015 | 0.097 |
| Homegrown tuber consumption (g kg^-1^ bw d^-1^) | 0.155 | 0.074 | 0.051 | -0.029 | 0.177 |
| Homegrown herbaceous fruit consumption(g kg^-1^ bw d^-1^) | 0.139 | -0.161 | 0.107 | -0.377 | 0.055 |
| *Non-homegrown shrub fruit consumption (g kg^-1^ bw d^-1^) | 0.010 | 0.135 | 0.050 | 0.033 | 0.236 |
| *Homegrown shrub fruit consumption (g kg^-1^ bw d^-1^) | 0.048 | 0.364 | 0.179 | 0.004 | 0.724 |
| Non-homegrown tree fruit consumption (g kg^-1^ bw d^-1^) | 0.036 | 0.056 | 0.026 | 0.004 | 0.108 |
| Soil lead concentration (mg kg^-1^) | 0.138 | -0.001 | 0.000 | -0.002 | 0.000 |

Notes: *significant predictor of BLL, **predictor of lower blood lead level

**The following variables were excluded after using stepwise model selection:** Smoking behaviours, hand to mouth behaviours, past or present activities with potential lead exposure, having pets, house hoovering or sweeping frequency, having current employment, fruit and vegetable washing behaviours, average daily amount of homegrown root vegetables eaten, average daily amount of tree fruit eaten, average daily amount of herbaceous fruit eaten, total number of years of being an allotment gardener, total number of years on current allotment, usual allotment visit length, usual allotment visit frequency, allotment in urban or rural location.

**Table SI5. Gardener only cohort: Results from the linear regression model showing predictors of log blood lead for the gardener only cohort using stepwise model selection.**

| **Predictors/Explanatory variables** | **p-value** | **Co- efficient** | **Standard error** | **95% confidence interval** | |
| --- | --- | --- | --- | --- | --- |
| *Age (years) | 0.002 | 0.070 | 0.014 | 0.037 | 0.103 |
| Gender: males compared to females | 0.218 | 0.211 | 0.156 | -0.158 | 0.580 |
| General health: bad compared to very good | 0.125 | -0.434 | 0.250 | -1.024 | 0.156 |
| *Alcohol consumption (unit) | 0.002 | 0.247 | 0.053 | 0.123 | 0.372 |
| *Previous smoker compared to never smoking | 0.052 | 0.439 | 0.188 | -0.005 | 0.883 |
| Current smoker compared to never smoking | 0.692 | 0.110 | 0.266 | -0.519 | 0.738 |
| Bite nails or similar: Yes compared to No | 0.404 | 0.195 | 0.220 | -0.325 | 0.716 |
| Tap water Pb concentrations (µg L^-1^) | 0.183 | 0.066 | 0.044 | -0.039 | 0.170 |
| **Lead pipe: run water compared to not run water | 0.003 | -1.306 | 0.299 | -2.014 | -0.598 |
| **No lead pipe compared to having lead pipe and not run water | 0.001 | -1.354 | 0.250 | -1.946 | -0.762 |
| **`Don’t know’ compared to having lead pipes and not running water | 0.042 | -0.800 | 0.322 | -1.561 | -0.038 |
| House vacuumed (frequency from every day vs less than once a month) | 0.397 | -0.044 | 0.049 | -0.160 | 0.072 |
| **House dusted (frequency from 5-6 times a week vs less than once a month) | 0.014 | 0.377 | 0.116 | 0.101 | 0.652 |
| *Employed compared to unemployed | 0.002 | 1.675 | 0.358 | 0.827 | 2.522 |
| Total number of years of being a UAS gardener | 0.383 | 0.018 | 0.020 | -0.028 | 0.065 |
| *Years at current UAS | 0.008 | 0.089 | 0.024 | 0.032 | 0.146 |
| Soil lead concentration (mg kg^-1^) | 0.060 | -0.001 | 0.001 | -0.003 | 0.000 |
| *Urban compared to rural UAS | 0.005 | 1.513 | 0.382 | 0.764 | 2.262 |
| UAS Visit frequency (from no visit to every day) | 0.150 | -0.184 | 0.114 | -0.454 | 0.085 |
| **^θ^UAS Visit length (from <1 to 4-6 hours) | 0.004 | -0.733 | 0.171 | -1.137 | -0.329 |
| *Washing fruit and veg: sometimes compared to usually | 0.017 | 1.083 | 0.350 | 0.256 | 1.910 |
| Washing fruit and veg: never compared to usually | 0.164 | 0.509 | 0.327 | -0.265 | 1.283 |
| **Non-homegrown green vegetable consumption (g kg^-1^ bw d^-1^) | 0.002 | -0.471 | 0.101 | -0.711 | -0.232 |
| **Non-homegrown herbaceous fruit consumption (g kg^-1^ bw d^-1^) | 0.034 | -0.157 | 0.060 | -0.299 | -0.015 |
| Non-homegrown root vegetable consumption (g kg^-1^ bw d^-1^) | 0.240 | 0.171 | 0.133 | -0.144 | 0.487 |
| Non-homegrown shrub fruit consumption (g kg^-1^ bw d^-1^) | 0.227 | 0.302 | 0.228 | -0.237 | 0.840 |
| *Non-homegrown tree fruit consumption (g kg^-1^ bw d^-1^) | 0.013 | 0.193 | 0.058 | 0.055 | 0.331 |
| Non-homegrown tuber consumption (g kg^-1^ bw d^-1^) | 0.214 | 0.049 | 0.036 | -0.036 | 0.133 |
| **Homegrown green vegetable consumption (g kg^-1^ bw d^-1^) | 0.017 | -0.256 | 0.082 | -0.450 | -0.062 |
| *Homegrown shrub fruit consumption (g kg^-1^ bw d^-1^) | 0.008 | 0.542 | 0.146 | 0.196 | 0.888 |
| **Homegrown herbaceous fruit consumption (g kg^-1^ bw d^-1^) | 0.008 | -0.346 | 0.095 | -0.569 | -0.122 |
| *Homegrown root vegetable consumption (g kg^-1^ bw d^-1^) | 0.048 | 0.349 | 0.146 | 0.004 | 0.694 |
| **Homegrown tree fruit consumption (g kg^-1^ bw d^-1^) | 0.024 | -0.465 | 0.162 | -0.847 | -0.083 |

Notes: *significant predictor of BLL, **predictor of lower blood lead level, ^θ^abnormal result

**The following variables were excluded after using stepwise model selection:** Average daily amount of homegrown root vegetables eaten, having pets and past or present activities with potential lead exposure.

**The following non-gardening variables are key for gardeners but not for controls:** Alcohol consumption (unit),

Having lead pipes and flushing through compared to not flushing through, house dusting frequency, going out to work, non-homegrown green vegetable consumption, non-homegrown herbaceous fruit consumption, non-homegrown tree fruit consumption.

| **Predictors/Explanatory variables** | **p-value** | **Co-efficient** | **Standard error** | **95% confidence interval** | |
| --- | --- | --- | --- | --- | --- |
| *Age (years) | 0.010 | 0.016 | 0.005 | 0.005 | 0.027 |
| Gender: males compared to females | 0.098 | -0.259 | 0.140 | -0.577 | 0.058 |
| *Lead activities : lots compared to non | 0.000 | 0.840 | 0.151 | 0.498 | 1.181 |
| *General health: bad compared to very good | 0.035 | 0.506 | 0.203 | 0.046 | 0.966 |
| *^θ^Previous smoker compared to never smoking | 0.029 | -0.360 | 0.139 | -0.675 | -0.046 |
| Current smoker compared to never smoking | 0.094 | 0.642 | 0.343 | -0.133 | 1.418 |
| Bite nails or similar: Yes compared to no | 0.094 | 0.279 | 0.149 | -0.058 | 0.617 |
| *Have pets: yes compared to no | 0.013 | 0.694 | 0.224 | 0.188 | 1.200 |
| Lead pipe: run water compared to not run water | 0.182 | 0.564 | 0.390 | -0.318 | 1.446 |
| No lead pipe compared to having lead pipe and not run water | 0.156 | -0.470 | 0.303 | -1.157 | 0.216 |
| `Don’t know’ compared to having lead pipe and not run water | 0.125 | -0.466 | 0.276 | -1.089 | 0.157 |
| Non-homegrown green vegetable consumption (g kg^-1^ bw d^-1^) | 0.153 | -0.098 | 0.063 | -0.239 | 0.044 |
| Non-homegrown herbaceous fruit consumption (g kg^-1^ bw d^-1^) | 0.358 | -0.091 | 0.093 | -0.302 | 0.121 |
| Non-homegrown root vegetable consumption (g kg^-1^ bw d^-1^) | 0.408 | 0.081 | 0.094 | -0.131 | 0.294 |
| *Non-homegrown shrub fruit consumption (g kg^-1^ bw d^-1^) | 0.035 | 0.200 | 0.081 | 0.017 | 0.382 |
| Non-homegrown tree fruit consumption (g kg^-1^ bw d^-1^) | 0.085 | 0.088 | 0.046 | -0.015 | 0.191 |
| Non-homegrown tuber consumption (g kg^-1^ bw d^-1^) | 0.214 | -0.131 | 0.098 | -0.353 | 0.091 |
| Alcohol consumption (unit) | 0.404 | -0.034 | 0.039 | -0.121 | 0.054 |

**Table SI 6. Results from the linear regression model showing predictors of log blood lead for the control only cohort using stepwise model selection.**

Notes: *significant predictor of BLL, **predictor of lower blood lead level, ^θ^Abnormal result

**The following variables were excluded after using stepwise model selection:** vacuuming frequency, tap water Pb concentration, house dusting frequency, and going out to work.

**Table SI 7. Demographic and behaviour predictors of higher and lower blood lead levels (BLL) indicated by this study**

| **Cohort Group** | **Predicts Lower BLL** | **Predicts Higher BLL** |
| --- | --- | --- |
| **All** | Eating more green vegetables (HG) | Being older |
|  | Eating more green vegetables (NHG) | Being male |
|  |  | Eating more root vegetables (NHG) |
|  |  | Eating more tubers (NHG) |
|  |  | Eating more shrub fruit (NHG) |
|  |  | Eating more tree fruit (NHG) |
|  |  | Eating more shrub (HG) |
| **Gardeners Only** | Eating more green vegetables (NHG) | Being older |
|  | Eating more green vegetables (HG) | Going out to work |
|  | Eating more herbaceous fruit (HG) | Drinking more alcohol |
|  | Eating more herbaceous fruit (NHG) | Having an urban allotment site |
|  | Eating more tree fruit (HG) | No lead pipes versus lead pipes not run =lower BLL |
|  | Dusting house more often | More years at the current allotment |
|  | Washing fruit and vegetables before eating | Making shorter visits to the allotment |
|  |  | Eating more shrub fruit (HG) |
|  |  | Eating more tree fruit (NHG) |
|  |  | Eating more root vegetables (HG) |
|  |  | Having lead pipes but not flushing out before collecting drinking or cooking water. |
| **Controls Only** |  | Being older |
|  |  | Undertaking more activities with potential lead exposure |
|  |  | Having bad health |
|  |  | Current smoker vs. Former & non smokers |
|  |  | Having cats or dogs |
|  |  | Eating more shrub fruit (NHG) |

Notes: NHG = Non home grown, HG = Homegrown

This table shows a list of behaviours and activities that are associated with either an increase or a decrease in blood lead levels. These effects are provided for controls only, gardeners only and for the groups combined.
